# The signal of ill-defined CPT weakening entanglement in the ${B_d}$ system

**DOI:** 10.1140/epjc/s10052-017-5432-2

**Published:** 2017-12-13

**Authors:** José Bernabéu, Francisco J. Botella, Nick E. Mavromatos, Miguel Nebot

**Affiliations:** 1 Departament de Física Teòrica, IFIC, Universitat de València-CSIC, 46100 Burjassot, Spain; 2 Theoretical Particle Physics and Cosmology Group, Department of Physics, King’s College London, Strand, London, WC2R 2LS UK; 3 Departamento de Física, Centro de Física Teórica de Partículas (CFTP), Instituto Superior Técnico (IST), U. de Lisboa (UL), Av. Rovisco Pais, 1049-001 Lisboa, Portugal

## Abstract

In the presence of quantum-gravity fluctuations (space-time foam), the CPT operator may be ill-defined. Its perturbative treatment leads to a modification of the Einstein–Podolsky–Rosen correlation of the neutral meson system by adding an entanglement-weakening term of the wrong exchange symmetry, the $\omega$-effect. In the current paper we identify how to probe the complex $\omega$ in the entangled $B_d$-system using the flavour (*f*)–CP(*g*) eigenstate decay channels: the connection between the intensities for the two time-ordered decays (*f*, *g*) and (*g*, *f*) is lost. Appropriate observables are constructed allowing independent experimental determinations of Re($\omega$) and Im($\omega$), disentangled from CPT violation in the evolution Hamiltonian Re($\theta$) and Im($\theta$). $2\sigma$ tensions for both Re($\theta$) and Im($\omega$) are shown to be uncorrelated.

## Introduction

The physics of discrete symmetries in particle and nuclear physics has always been a fascinating subject, since the observation of CP violation in the neutral kaon system [1], which was a clear experimental surprise and set the scene for subsequent precision tests of such discrete symmetries in other systems, including entangled neutral meson factories. Today CP violation in the *K* and $B_d$ systems, as well as T violation with entangled $B_d$’s [2], have been demonstrated experimentally to great accuracy. However, their combination CPT remains unbroken. This is believed to be due to one of the crucial theorems of modern physics, ensuring CPT Invariance of quantum field theory models that are Lorentz invariant, local (in their interactions) and unitary (that is, they conserve probability) [3]. This is basically a theorem of flat space-time. Quantum gravity or in general deviations from any of the three assumptions may lead to (independent) violations of CPT, which, if observed in nature, would undoubtedly constitute an indication of completely novel physics. Having mentioned quantum gravity, it is worth recalling a corollary by Wald [4], according to which a potential decoherence induced during observations in local scattering experiments in which the experimenter has no access to microscopic quantum gravity degrees of freedom, may lead to an effectively ill-defined CPT quantum mechanical operator. This observation prompted the authors of [5] to introduce a different observable for this kind of decoherence-induced CPT violation, termed $\omega$-effect. The $\omega$-effect is different from the situation where CPT violation is violated in the effective Hamiltonian, parameterized by the complex $\theta$ parameter. Among other possible sources, $\theta$ can be due to, e.g., Lorentz violation [6, 7] as a result of propagation in some Lorentz violating space-time (or otherwise) backgrounds. In the latter case the quantum mechanical operator that implements CPT symmetry is well defined but simply does not commute with the Hamiltonian. The $\omega$-effect, if observed, points to an observation of a phenomenon that is exclusively linked to ill-defined nature of the CPT operator, which to date is theoretically linked only to fundamental decoherence [4], independently of any violation of CPT in the Hamiltonian.

Recently, a study for separate direct evidence of T, CP, CPT symmetry violation was accomplished [8]. It was based on the precise identification of genuine asymmetry parameters in the time evolution of intensities between the two decays in a B-Factory of entangled neutral $B_d$-meson states. Their values were obtained from the BaBar measurements [2] of the different flavour-CP eigenstate decay channels. The concept, put forward in [9, 10], uses the entangled character of the initial state as the crucial ingredient to (i) connect experimental double decay rates with specific meson transition probabilities and (ii) identify the transformed transition to that taken as a reference [11, 12]. Possible fake effects [8] were demonstrated to be well under control by measurements in the same experiment. The methodology, discussed in [9, 10], appears to be [13] crucially dependent on the assumed maximal entanglement between $B^0_d$ and $\bar{B}^0_d$, or between two orthogonal superpositions of them, as given by the Einstein–Podolsky–Rosen (EPR) correlation [14] imposed by their decay from the $\varUpsilon (4S)$-state with $C = -$. The corresponding antisymmetric state of the system has two important implications: (i) the program of using entanglement and the decays as filtering measurements to prepare and detect the meson states can be implemented at any time for the first decay, even in the presence of mixing during the previous entangled evolution; (ii) the coefficients of the different time-dependent terms in the double decay rate intensities for the time-ordered decays to (*g*, *f*) are related to those for the time-ordered decays to (*f*, *g*). The antisymmetry of the entangled state is kept for any two independent states of the neutral mesons, so its evolution leads to a trivial time dependence with definite symmetry under the combined exchange $(f,t_0; g,t_0+t)\rightarrow (g,t_0-t;f,t_0)$. As a consequence, the double decay rate intensity (see Eq. 14 below) satisfies for the coefficients of its time dependence with $\omega =0$,

1 $$\begin{aligned} \mathscr {C}_{h}[f,g]= & {} \mathscr {C}_{h}[{g},{f}],\quad \mathscr {C}_{c}[{f},{g}] =\mathscr {C}_{c}[g,f]\nonumber \\&\quad \text {and}\quad \mathscr {S}_{c}[f,g]=-\mathscr {S}_{c}[g,f], \end{aligned}$$

where the time-ordered decays (*f*, *g*) and (*g*, *f*) are, in general, not connected by any symmetry transformation. At this level, they can be considered as two different experimental ways of measuring the same quantity when $\omega =0$.

In the application to definite flavour or CP eigenstates decay products, the preparation by maximal entanglement of the initial state of a single neutral meson is usually referred to as “flavour tagging” $B^0_d$, $\bar{B}^0_d$, or “CP tagging” $B_+$, $B_-$. The underlying assumption considers $B^0_d$, $\bar{B}^0_d$ as two states of the same field, in order to impose Bose statistics with charge conjugation C and permutation $\mathcal P$ with C$\mathcal P$ $= +$, and it may be invalidated if the CPT operator cannot be intrinsically well defined, as mentioned above. This latter circumstance may occur, for example, in the context of an extended class of quantum-gravity models, where the structure of quantum space-time at Planckian scales ($10^{-35}$ m) may actually be fuzzy, characterized by a “foamy” nature (space-time foam) [5, 15, 16]. Let us emphasize once more that this kind of CPT breaking is different from an explicit CPT violation in the Hamiltonian dynamics such that [CPT, H] $\ne 0$, as conventionally introduced, in the context of the Weisskopf–Wigner approach [17–19] for the neutral meson system, in the mass matrix. This last CPT violation does not invalidate the analysis followed in [8] and, in fact, genuine observables for CPT violation were found with their values obtained from experiment. However, the CPT breaking associated to “ill-defined” particle–antiparticle states modifies the EPR correlation, producing the aforementioned $\omega$-effect [5, 20, 21]. Treating it in perturbation theory, in such a way that we still talk the language of $B^0_d$, $\bar{B}^0_d$, the perturbed two-particle state will contain a component of the “wrong” symmetry at the instant of their production by the decay of $\varUpsilon (4S)$:

2 $$\begin{aligned} |\varPsi _0\rangle \propto |B^0_d\rangle |\bar{B}^0_d\rangle -|\bar{B}^0_d\rangle |B^0_d\rangle +\omega [|B^0_d\rangle |\bar{B}^0_d\rangle +|\bar{B}^0_d\rangle |B^0_d\rangle ],\nonumber \\ \end{aligned}$$

where $\omega = |\omega | \mathrm{e}^{i\varOmega }$ is a complex CPT-breaking parameter [5, 20], associated with the non-identical particle nature of the neutral meson and antimeson states. The presence of an $\omega$-effect weakens the entanglement of the initial state (2), as follows from the fact that when $\omega =\pm 1$ the state simply reduces to a product state, whilst when $\omega =0$ the state is fully entangled.

We emphasize that the modification in Eq. (2) is due to the loss of indistinguishability of $B^0_d$ and $\bar{B}^0_d$ and not due to violation of symmetries in the production process. Evidently, the probabilities for the two states connected by a permutation are different due to the presence of $\omega$. This modification of the initial state vector has far-reaching consequences for the concept of meson tagging and for the relation of the time-dependent intensities between the decays to time-ordered (*f*, *g*) and (*g*, *f*) channel.^1^

In what follows we will study the non-trivial time evolution of Eq. (2), in the simplified but physically relevant case of a time-independent $\omega$, in order to (i) establish the appearance of terms of the (previously forbidden) type $|B^0_d\rangle |B^0_d\rangle$ and $|\bar{B}^0_d\rangle |\bar{B}^0_d\rangle$, and (ii) introduce a set of observables, which actually serve as a direct way for measuring $\omega$, based on the violation of the relations of Eq. (1), i.e. using as observables for $\omega \ne 0$:

4 $$\begin{aligned}&\mathscr {C}_{h}^\omega [f,g]-\mathscr {C}_{h}^\omega [g,f],\quad \mathscr {C}_{c}^\omega [f,g] -\mathscr {C}_{c}^\omega [g,f]\nonumber \\&\quad \quad \text {and}\quad \mathscr {S}_{c}^\omega [f,g]+\mathscr {S}_{c}^\omega [g,f], \end{aligned}$$

and checking experimentally the robustness of the correlation between the two states assumed during the tagging. This paper demonstrates that the comparison between the double decay rate Intensities for time-ordered ($f=$ flavour, $g=$ CP) eigenstate decay products and (*g*, *f*) is sensitive to both $\text {Re}\left( \omega \right)$ and $\text {Im}\left( \omega \right)$.

## Time evolution

### Double decay rates, time-dependent intensities

The eigenstates of the effective Hamiltonian $\mathbf {H}$ are^2^

5 $$\begin{aligned} \begin{aligned}&\mathbf {H}|B_H\rangle =\mu _H|B_H\rangle ,\quad |B_H\rangle =p_H|B^0_d\rangle +q_H|\bar{B}^0_d\rangle ,\\&\mathbf {H}|B_L\rangle =\mu _L|B_L\rangle ,\quad \ |B_L\rangle =p_L|B^0_d\rangle -q_L|\bar{B}^0_d\rangle . \end{aligned} \end{aligned}$$

In terms of these

6 $$\begin{aligned}&|\varPsi _0\rangle \propto |B_L\rangle |B_H\rangle -|B_H\rangle |B_L\rangle \nonumber \\&\quad +\,\omega \Biggl \{\theta [|B_H\rangle |B_L\rangle +|B_L\rangle |B_H\rangle ]\nonumber \\&\quad +\,(1-\theta )\frac{p_L}{p_H}|B_H\rangle |B_H\rangle -(1+\theta )\frac{p_H}{p_L}|B_L\rangle |B_L\rangle \Biggr \}, \end{aligned}$$

where $\theta$ is a CP and CPT violating complex parameter given by $\theta =\frac{\mathbf {H}_{22}-\mathbf {H}_{11}}{\mu _H-\mu _L}$. The time evolution of two-meson flavour states is

7 $$\begin{aligned}&\begin{pmatrix} |\mathrm {A}(t)\rangle \\ |B^0_d(t)\rangle |B^0_d(t)\rangle \\ |\mathrm {S}(t)\rangle \\ |\bar{B}^0_d(t)\rangle |\bar{B}^0_d(t)\rangle \end{pmatrix}\nonumber \\&\quad = \mathrm{e}^{-\varGamma \,t}\mathrm{e}^{-i2M\,t} \begin{pmatrix} 1&\begin{matrix} 0 &{}\,&{} &{} 0 &{}\,&{} &{} 0 \end{matrix}\\ \begin{matrix} 0 \\ 0 \\ 0 \end{matrix}&\mathrm {C} + \mathrm {E}_{[+]} \mathrm{e}^{i\varDelta \mu \,t}+\mathrm {E}_{[-]} \mathrm{e}^{-i\varDelta \mu \,t} \end{pmatrix} \begin{pmatrix} |\mathrm {A}\rangle \\ |B^0_d\rangle |B^0_d\rangle \\ |\mathrm {S}\rangle \\ |\bar{B}^0_d\rangle |\bar{B}^0_d\rangle \end{pmatrix},\nonumber \\ \end{aligned}$$

where $$\mu _H+\mu _L=2M-i\varGamma $$, $$\mu _H-\mu _L=\varDelta \mu =\varDelta M-i\frac{\varDelta \varGamma }{2}$$,

8 $$\begin{aligned} \begin{matrix} |\mathrm {A}(t)\rangle =\frac{1}{\sqrt{2}}\left[ |B^0_d(t)\rangle |\bar{B}^0_d(t)\rangle -|\bar{B}^0_d(t)\rangle |B^0_d(t)\rangle \right] ,\\ |\mathrm {S}(t)\rangle =\frac{1}{\sqrt{2}}\left[ |B^0_d(t)\rangle |\bar{B}^0_d(t)\rangle +|\bar{B}^0_d(t)\rangle |B^0_d(t)\rangle \right] , \end{matrix} \end{aligned}$$

and

9 $$\begin{aligned} |B^0_d(t)\rangle =\mathrm{e}^{-i\mathbf {H}t}\,|B^0_d\rangle ,\quad |\bar{B}^0_d(t)\rangle =\mathrm{e}^{-i\mathbf {H}t}\,|\bar{B}^0_d\rangle . \end{aligned}$$

The $\mathrm {C}$, $\mathrm {E}_{[\pm ]}$ matrices are

10 $$\begin{aligned}&\mathrm {C}= \begin{pmatrix} \frac{1}{2}(1-\theta ^2) &{} \frac{1}{\sqrt{2}}\frac{q}{p}\theta \sqrt{1-\theta ^2} &{} -\frac{1}{2}\frac{q^2}{p^2}(1-\theta ^2)\\ \frac{1}{\sqrt{2}}\frac{p}{q}\theta \sqrt{1-\theta ^2} &{} \theta ^2 &{} -\frac{1}{\sqrt{2}}\frac{q}{p}\theta \sqrt{1-\theta ^2}\\ -\frac{1}{2}\frac{p^2}{q^2}(1-\theta ^2) &{} -\frac{1}{\sqrt{2}}\frac{p}{q}\theta \sqrt{1-\theta ^2} &{} \frac{1}{2}(1-\theta ^2) \end{pmatrix}, \end{aligned}$$

11 $$\begin{aligned}&\mathrm {E}_{[+]}= \begin{pmatrix} \frac{1}{4}(1+\theta )^2 &{} -\frac{1}{2\sqrt{2}}\frac{q}{p}(1+\theta )\sqrt{1-\theta ^2} &{} \frac{1}{4}\frac{q^2}{p^2}(1-\theta ^2)\\ -\frac{1}{2\sqrt{2}}\frac{p}{q}(1+\theta )\sqrt{1-\theta ^2} &{} \frac{1}{2}(1-\theta ^2) &{} -\frac{1}{2\sqrt{2}}\frac{q}{p}(1-\theta )\sqrt{1-\theta ^2}\\ \frac{1}{4}\frac{p^2}{q^2}(1-\theta ^2) &{} -\frac{1}{2\sqrt{2}}\frac{p}{q}(1-\theta )\sqrt{1-\theta ^2} &{} \frac{1}{4}(1-\theta )^2 \end{pmatrix}, \end{aligned}$$

12 $$\begin{aligned}&\mathrm {E}_{[-]}= \begin{pmatrix} \frac{1}{4}(1-\theta )^2 &{} \frac{1}{2\sqrt{2}}\frac{q}{p}(1-\theta )\sqrt{1-\theta ^2} &{} \frac{1}{4}\frac{q^2}{p^2}(1-\theta ^2)\\ \frac{1}{2\sqrt{2}}\frac{p}{q}(1-\theta )\sqrt{1-\theta ^2} &{} \frac{1}{2}(1-\theta ^2) &{} \frac{1}{2\sqrt{2}}\frac{q}{p}(1+\theta )\sqrt{1-\theta ^2}\\ \frac{1}{4}\frac{p^2}{q^2}(1-\theta ^2) &{} \frac{1}{2\sqrt{2}}\frac{p}{q}(1+\theta )\sqrt{1-\theta ^2} &{} \frac{1}{4}(1+\theta )^2 \end{pmatrix}. \end{aligned}$$

In Eqs. (10)–(12), $\frac{q}{p}$ is the usual meson mixing quantity given by $\frac{q^2}{p^2}=\frac{\mathbf {H}_{21}}{\mathbf {H}_{12}}=\frac{q_Hq_L}{p_Hp_L}$. Before addressing actual observables, it is worth noting that, considering Eq. (7), it is clear that the presence of the symmetric state $|\mathrm {S}\rangle$ in Eq. (2) induces the appearance of $|B^0_d\rangle |B^0_d\rangle$ and $|\bar{B}^0_d\rangle |\bar{B}^0_d\rangle$ states.

The transition amplitude for the decay of the first state into $|f\rangle$ at time $t_{0}$, and then the second state into $|g\rangle$ at time $t+t_{0}$ is $\langle f,t_0;g,t+t_0|T|\varPsi _0\rangle$. Squaring and integrating over $t_{0}$, the double decay rate *I*(*f*, *g*; *t*) is obtained:

13 $$\begin{aligned} I(f,g;t)=\int _0^\infty \mathrm{d}t_0\,|\langle f,t_0;g,t+t_0|T|\varPsi _0\rangle |^2. \end{aligned}$$

Expanding to first order in $\omega$, $\theta$ and taking $\varDelta \varGamma =0$, *I*(*f*, *g*; *t*) has the following form for generic *f* and *g* decay channels^3^:

14 $$\begin{aligned} I(f,g;t)= & {} \frac{\langle \varGamma _f\rangle \langle \varGamma _g\rangle }{\varGamma }\mathrm{e}^{-\varGamma \,t} \{ \mathscr {C}_{h}^\omega [f,g]\nonumber \\&+\,\mathscr {C}_{c}^\omega [f,g]\cos (\varDelta Mt)+\mathscr {S}_{c}^\omega [f,g]\sin (\varDelta Mt) \},\nonumber \\ \end{aligned}$$

with

15 $$\begin{aligned} \mathscr {C}_{h}^\omega [f,g]= & {} N_{[f,g]}\Biggl [ 1-R_{f}R_{g}+\text {Re}\left( \theta \right) (C_{g}R_{f}+C_{f}R_{g})\nonumber \\&-\,\text {Im}\left( \theta \right) (S_{f}+S_{g})\nonumber \\&+\frac{1}{1+(x/2)^2} \big \{ (2C_{f}+xS_{f})\text {Re}\left( \omega \right) \nonumber \\&+(xC_{f}-2S_{f})R_{g}\text {Im}\left( \omega \right) \big \} \Biggr ], \end{aligned}$$

16 $$\begin{aligned} \mathscr {C}_{c}^\omega [f,g]= & {} N_{[f,g]}\Biggl [ -(C_{f}C_{g}+S_{f}S_{g})\nonumber \\&-\,\text {Re}\left( \theta \right) (C_{g}R_{f}+C_{f}R_{g})\nonumber \\&+\,\text {Im}\left( \theta \right) (S_{f}+S_{g})\nonumber \\&+\,\frac{1}{1+(x/2)^2} \big \{ -(2C_{g}+xS_{g})\text {Re}\left( \omega \right) \nonumber \\&+\,(-xC_{g}+2S_{g})R_{f}\text {Im}\left( \omega \right) \big \} \Biggr ], \end{aligned}$$

17 $$\begin{aligned} \mathscr {S}_{c}^\omega [f,g]= & {} N_{[f,g]}\Biggl [ (C_{g}S_{f}-C_{f}S_{g})\nonumber \\&+\,\text {Re}\left( \theta \right) (R_{g}S_{f}-R_{f}S_{g})\nonumber \\&+\,\text {Im}\left( \theta \right) (C_{f}-C_{g})\nonumber \\&+\,\frac{1}{1+(x/2)^2} \big \{ (xC_{g}-2S_{g})\text {Re}\left( \omega \right) \nonumber \\&-\,(2C_{g}+xS_{g})R_{f}\text {Im}\left( \omega \right) \big \} \Biggr ], \end{aligned}$$

where, in terms of the decays amplitudes $\langle f|T|\bar{B}^0_d\rangle \equiv \bar{A}_f$ and $\langle f|T|B^0_d\rangle \equiv A_f$, the following parameters are used^4^:

18 $$\begin{aligned}&\lambda _{f}\equiv \frac{q}{p}\frac{\bar{A}_f}{A_f},\quad C_{f}=\frac{1-|\lambda _{f}|^2}{1+|\lambda _{f}|^2},\quad R_{f}= \frac{2\text {Re}\left( \lambda _{f}\right) }{1+|\lambda _{f}|^2},\quad \nonumber \\&S_{f}= \frac{2\text {Im}\left( \lambda _{f}\right) }{1+|\lambda _{f}|^2}, \end{aligned}$$

19 $$\begin{aligned}&N_{[f,g]}=\frac{1-\delta ^2}{(1+|\omega |^2)(1-\delta C_{f})(1-\delta C_{g})},\nonumber \\&\text {and }\langle \varGamma _f\rangle =\frac{|\bar{A}_f|^2+|A_f|^2}{2}. \end{aligned}$$

In addition, $x=\frac{\varDelta M}{\varGamma }\simeq 0.77$ and $\delta =\frac{1-|q/p|^2}{1+|q/p|^2}\simeq 1-2\times 10^{-3}$. It is worth recalling that, for the flavour-specific decay channels $X+\ell ^\pm$ (“$\ell ^\pm$” for short in the following), we have $C_{\ell ^\pm }=\pm 1$, $R_{\ell ^\pm }=S_{\ell ^\pm }=0$.

### Sensitivity to ${\omega }$

Coming back to the transition amplitude $\langle f,t_0;g,t+t_0|T|\varPsi _0\rangle$, it has the following structure:

20 $$\begin{aligned}&\langle f,t_0;g,t+t_0|T|\varPsi _0\rangle \propto \mathrm{e}^{(-iM-\varGamma /2)(2t_0+t)}\nonumber \\&\quad \begin{pmatrix} [\mathrm{e}^{-i\varDelta \mu \,t/2}\mathcal A_{f}^{L}\mathcal A_{g}^{H}-\mathrm{e}^{i\varDelta \mu \,t/2}\mathcal A_{f}^{H}\mathcal A_{g}^{L}]\\ +\omega \theta [\mathrm{e}^{-i\varDelta \mu \,t/2}\mathcal A_{f}^{L}\mathcal A_{g}^{H}+\mathrm{e}^{i\varDelta \mu \,t/2}\mathcal A_{f}^{H}\mathcal A_{g}^{L}]\\ +\omega (1-\theta )\frac{p_L}{p_H}\mathrm{e}^{-i\varDelta \mu \,(t_0+t/2)}\mathcal A_{f}^{H}\mathcal A_{g}^{H}\\ -\omega (1+\theta )\frac{p_H}{p_L}\mathrm{e}^{i\varDelta \mu \,(t_0+t/2)}\mathcal A_{f}^{L}\mathcal A_{g}^{L} \end{pmatrix}. \end{aligned}$$

The prefactor $\mathrm{e}^{(-iM-\varGamma /2)(2t_0+t)}$ gives a global $\mathrm{e}^{-2\varGamma t_0}\mathrm{e}^{-\varGamma t}$ dependence in $|\langle f,t_0;g,t+t_0|T|\varPsi _0\rangle |^2$. One can readily observe that the $\omega$-dependent terms, even for $\theta =0$ (i.e. already for the leading $\omega$ contribution), do introduce an additional non-trivial $t_0$ dependence. Ignoring that $\mathrm{e}^{(-iM-\varGamma /2)(2t_0+t)}$ prefactor, it is clear that combining the transformations $t\mapsto -t$ and $f\leftrightarrows g$, the first contribution, the standard $\omega =0$ one, just receives a $(-)$ sign. This implies that, in the absence of $\omega$, in the *t*-dependence of *I*(*f*, *g*; *t*),

21 $$\begin{aligned}&I(f,g;t)\sim \mathrm{e}^{-\varGamma t}\left( \mathscr {C}_{h}[f,g]+\mathscr {C}_{c}[f,g]\cos (\varDelta Mt)\right. \nonumber \\&\quad \left. +\,\mathscr {S}_{c}[f,g]\sin (\varDelta Mt)\right) \end{aligned}$$

we necessarily have [8]: $\mathscr {C}_{h}[f,g]=\mathscr {C}_{h}[g,f]$, $\mathscr {C}_{c}[f,g]=\mathscr {C}_{c}[g,f]$ and $\mathscr {S}_{c}[f,g]=-\mathscr {S}_{c}[g,f]$.

In the presence of $\omega \ne 0$ the situation changes drastically. From the remaining contributions in Eq. (20), the ones induced by the evolution of the $\omega$-dependent term in Eq. (2), the situation is more involved: the first one, proportional to $\omega \theta$ and $t_0$-independent, is clearly invariant under the combination of $f\leftrightarrows g$ and $t\mapsto -t$. The last two terms are separately invariant under $f\leftrightarrows g$, but have no well-defined transformation under $t\mapsto -t$; moreover, contrary to the previous contributions, they depend on $t_0$, the time elapsed between production of the $B\bar{B}$ pair and the first decay.^5^ Out of those properties, the simple assignment of symmetry/antisymmetry under $f\leftrightarrows g$ to the *t*-even/*t*-odd terms in $\mathrm{e}^{\varGamma \,t}\,I(f,g;t)$, possible when $\omega =0$, does not apply when $\omega \ne 0$. This simple remark provides the first understanding of the potential sensitivity to the presence of $\omega \ne 0$: while in the absence of $\omega$, the measurement of intensities for decays into *f* and *g* with the two different orderings (i) first *f* then *g* and (ii) first *g* then *f*, provides two experimentally independent measurements of the same theoretical quantities, in the presence of $\omega$ the situation has changed. Deviations from the standard $f\leftrightarrows g$ symmetry properties are a gateway to probe for $\omega$.

The BaBar collaboration performed separate analyses [2] for the two different time orderings of the two *B* meson decays. Previous studies, like [21], exploited the use of two flavour specific decay channels to obtain bounds on $\text {Re}\left( \omega \right)$ through the appearance of $|B^0_d\rangle |B^0_d\rangle$ and $|\bar{B}^0_d\rangle |\bar{B}^0_d\rangle$ states for $t=0$. Equation (14) shows that, using flavour specific channels alone, there is no sensitivity to $\text {Im}\left( \omega \right)$: since $R_{\ell ^\pm }=0$, the terms in $\text {Im}\left( \omega \right)$ would be absent.^6^ Fortunately enough, besides addressing the two different time orderings, in [2], one decay is flavour specific (labeled $\ell ^\pm$), while the other is CP specific (decays into $J/\varPsi K_{S,L}$, labeled $K_{S,L}$ for short): sensitivity to both $\text {Re}\left( \omega \right)$ and $\text {Im}\left( \omega \right)$ is thus expected.

### Experimental observables

In order to reduce experimental uncertainties in the different channels, the BaBar collaboration, in Ref. [2], fixed the constant term and measured the coefficients $\mathrm C[f,g]$ and $\mathrm S[f,g]$ of the decay intensity

22 $$\begin{aligned}&\mathbf {g}_{f,g}(t)\propto \mathrm{e}^{-\varGamma \, t}\left\{ 1+\mathrm C[f,g]\cos (\varDelta M\,t)\right. \nonumber \\&\quad \left. +\,\mathrm S[f,g]\sin (\varDelta M\,t)\right\} , \end{aligned}$$

using for the *f* and *g* states one flavour specific channel, $X\ell ^+\nu$ or $X\ell ^-\bar{\nu }$, and one CP eigenstate, $J/\varPsi K_S$ or $J/\varPsi K_L$. Obviously we should have

23 $$\begin{aligned} \mathrm C[f,g]=\frac{\mathscr {C}_{c}^\omega [f,g]}{\mathscr {C}_{h}^\omega [f,g]}\quad \text {and}\quad \mathrm S[f,g]=\frac{\mathscr {S}_{c}^\omega [f,g]}{\mathscr {C}_{h}^\omega [f,g]}, \end{aligned}$$

where one should remember that in the coefficients $\mathrm C[f,g]$ and $\mathrm S[f,g]$, the ordering of *f* and *g* means that *f* corresponds to the first (in time) decay product of the entangled state evolved in time, and *g* corresponds to the second (in time) decay product. In the case under consideration, the flavour specific decays simplify significantly the expressions, which are, at linear order in $\theta$, $\omega$,

24 $$\begin{aligned} \mathrm C[\ell ^\pm ,g]= & {} \mp C_{g}+\text {Re}\left( \theta \right) R_{g}(C_{g}\mp 1)+\text {Im}\left( \theta \right) S_{g}(1\mp C_{g})\nonumber \\&+\,\frac{1}{1+(x/2)^2}\{ -xS_{g}\text {Re}\left( \omega \right) +xC_{g}R_{g}\text {Im}\left( \omega \right) \}, \end{aligned}$$

25 $$\begin{aligned} \mathrm S[\ell ^\pm ,g]= & {} \mp S_{g}+\text {Re}\left( \theta \right) S_{g}R_{g}+\text {Im}\left( \theta \right) (\pm 1-C_{g}\mp S_{g}^2)\nonumber \\&+\,\frac{1}{1+(x/2)^2}\{ xC_{g}\text {Re}\left( \omega \right) +xS_{g}R_{g}\text {Im}\left( \omega \right) \}.\nonumber \\ \end{aligned}$$

In the presence of $\omega$, the time ordering definite symmetry is not valid anymore and therefore it is relevant to write the completely different coefficients

26 $$\begin{aligned} \mathrm C[f,\ell ^\pm ]= & {} \mp C_{f}+\text {Re}\left( \theta \right) R_{f}(C_{f}\mp 1)\nonumber \\&+\,\text {Im}\left( \theta \right) S_{f}(1\mp C_{f})\nonumber \\&+\,\frac{1}{1+(x/2)^2}\{ \pm (2(C_{f}^2-1)\nonumber \\&+\,xC_{f}S_{f}))\text {Re}\left( \omega \right) \mp xR_{f}\text {Im}\left( \omega \right) \}, \end{aligned}$$

27 $$\begin{aligned} \mathrm S[f,\ell ^\pm ]= & {} \pm S_{f}-\text {Re}\left( \theta \right) S_{f}R_{f}+\text {Im}\left( \theta \right) (\mp 1+C_{f}\pm S_{f}^2)\nonumber \\&+\,\frac{1}{1+(x/2)^2}\{ \pm (x(1-S_{f}^2)\nonumber \\&-\,2C_{f}S_{f})\text {Re}\left( \omega \right) \mp 2R_{f}\text {Im}\left( \omega \right) \}. \end{aligned}$$

As anticipated, $\mathrm C[\ell ^\pm ,g]-\mathrm C[g,\ell ^\pm ]$ and $\mathrm S[\ell ^\pm ,g]+\mathrm S[g,\ell ^\pm ]$ are linear in $\omega$, and thus the fact that the BaBar collaboration distinguished the different decay time orderings in [2], now reveals crucial to disentangle the $\omega$ effect:

28 $$\begin{aligned}&\mathrm C[\ell ^\pm ,g]-\mathrm C[g,\ell ^\pm ]\nonumber \\&\quad = \frac{1}{1+(x/2)^2} \{ [xS_{g}\mp 2(C_{g}^2-1)\mp xC_{g}S_{g}]\text {Re}\left( \omega \right) \nonumber \\&\qquad +\,xR_{g}[C_{g}\pm 1]\text {Im}\left( \omega \right) \}, \end{aligned}$$

29 $$\begin{aligned}&\mathrm S[\ell ^\pm ,g]+\mathrm S[g,\ell ^\pm ]\nonumber \\&\quad = \frac{1}{1+(x/2)^2} \{ [xC_{g}\pm x(1-S_{g}^2)\mp 2C_{g}S_{g}]\text {Re}\left( \omega \right) \nonumber \\&\qquad +\,R_{g}[xS_{g}\mp 2]\text {Im}\left( \omega \right) \}. \end{aligned}$$

These combinations are linearly sensitive not only to $\text {Re}\left( \omega \right)$ but also to $\text {Im}\left( \omega \right)$ when $R_{g}\ne 0$. The sensitivity to $\text {Im}\left( \omega \right)$ depends critically on the use of a CP eigenstate channel with large $R_{g}$, as is the case with $J/\varPsi K_S$ and $J/\varPsi K_L$.

Before we present our numerical results it is important to summarize the assumptions we have made in our analysis. In the $\omega =0$ limit, as explained after Eq. (21), we also have the set of equations

30 $$\begin{aligned} \mathscr {C}_{c}[f,g]-\mathscr {C}_{c}[g,f]=0,\quad \mathscr {S}_{c}[f,g]+\mathscr {S}_{c}[g,f]=0, \end{aligned}$$

therefore it is clear that any deviation from those zeros, as identified by Eqs. (28) and (29), will give a positive signal for $\omega \ne 0$. This result is independent of any additional assumption as regards the decay amplitudes, it is valid even if we consider any pair of decay channels *f* and *g*. This should not come as a surprise because we compare two double decay rates to the same final states: these observables only reverse the order in time of the “same” decay channels and the results are dictated by the symmetry properties of the initial state entering Eq. (20).

Independently of the previous remark it is important to stress that in the present analysis there are no assumptions on the decay amplitudes in the channels $B_d\rightarrow J/\varPsi K_{S,L}$. We follow here the parameterisation used in Ref. [8]. In that work, in order to point out genuine T violating observables independently of any assumption as regards CP or CPT, one is forced to allow for CPT violation both in the $B^0_d$–$\bar{B}^0_d$ mixing and in the $B_d\rightarrow J/\varPsi K_{S,L}$ decay amplitudes. One also needs to have a completely general parameterisation in order to measure any possible contamination coming from these contributions and others explained in [8]. Therefore we have parameterized $\lambda _{K_S}$ and $\lambda _{K_L}$ in terms of two arbitrary complex numbers that later on become four real arbitrary parameters $C_{K_S}$, $S_{K_S}$, $C_{K_L}$ and $S_{K_L}$ to be extracted from the fit itself ($R_{K_S}$ and $R_{K_L}$ only add two signs). This parameterisation fits any New Physics including Standard Model penguins, New Physics models or CPT violation in the decay amplitudes. Nevertheless we have worked under the following approximations, which we deem to be physically reasonable: (i) we have made the $\varDelta \varGamma _{B_d}=0$ approximation in order to use the Babar data, (ii) we have not allowed for wrong sign decays in flavour specific channels $B_d\rightarrow X+\ell ^\pm$ with the following implications: $C_{\ell ^\pm }=\pm 1$, $S_{\ell ^\pm }=R_{\ell ^\pm }=0$ as indicated at the end of Sect. 2.1. Note that this last assumption does not invalidate the fact that a difference between $\mathscr {C}_{c}[f,g]$ and $\mathscr {C}_{c}[g,f]$ (or between $\mathscr {S}_{c}[f,g]$ and $-\mathscr {S}_{c}[g,f]$) would be an unambiguous signal of $\omega \ne 0$.

## Results

We are now ready to present the results obtained from a global fit to available BaBar experimental data, following the same statistical treatment as in Ref. [8]. We use the 16 experimental observables measured by BaBar in [2]: $\mathrm C[\ell ^\pm ,K_{S,L}]$, $\mathrm C[K_{S,L},\ell ^\pm ]$, $\mathrm S[\ell ^\pm ,K_{S,L}]$ and $\mathrm S[K_{S,L},\ell ^\pm ]$. Taking into account full covariance information on statistical and systematic uncertainties, we perform a fit in terms of the set of parameters $\{\text {Re}\left( \theta \right)$, $\text {Im}\left( \theta \right)$, $\text {Re}\left( \omega \right)$, $\text {Im}\left( \omega \right)$, $C_{K_S}$, $S_{K_S}$, $R_{K_S}$, $C_{K_L}$, $S_{K_L}$, $R_{K_L}\}$ with the known constraints $C_{f}^2+S_{f}^2+R_{f}^2=1$. Therefore we generalize the corresponding fit presented in Ref. [8] to the actual situation where deviations from EPR entanglement are present due to the $\omega$-effect [5]. A more restricted fit is also done in the case where no wrong sign flavour decays are allowed in the $B_d\rightarrow J/\varPsi K$ decays, that is with $\lambda _{K_S}+\lambda _{K_L}=0$.

**Fig. 1:**
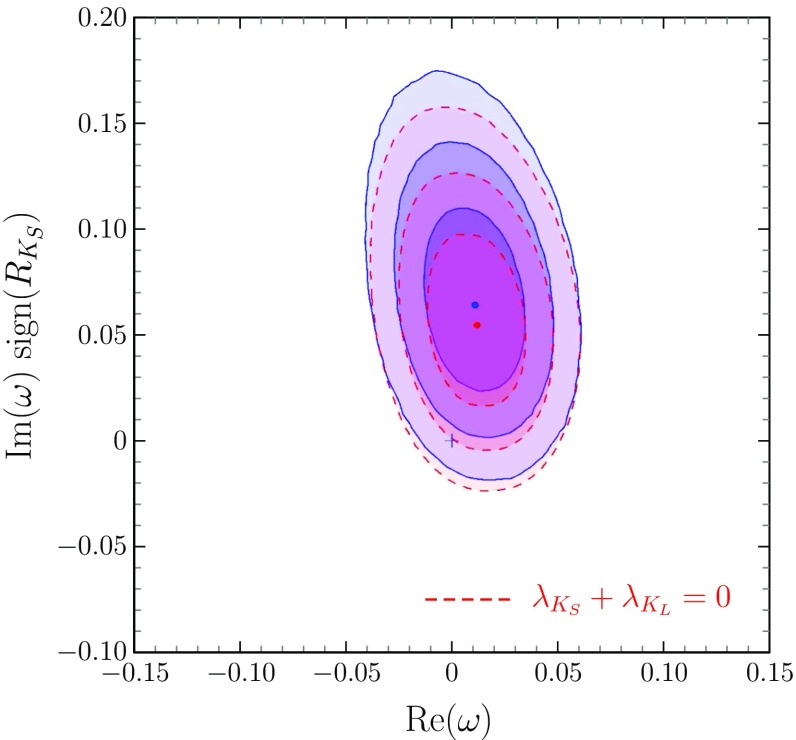
$\text {Im}\left( \omega \right)$ vs. $\text {Re}\left( \omega \right)$ in the general fit (blue regions with solid contours), and in the fit with $\lambda _{K_S}+\lambda _{K_L}=0$ (red regions with dashed contours); darker to lighter regions correspond to two-dimensional 68, 95 and 99% CL. Figures 3 and 4 obey the same colour coding for the two fits and the CL regions

**Table 1:** Summary of results

(I) Parameters – general analysis			
$\text {Re}\left( \theta \right)$	$\pm (6.11\pm 3.45)10^{-2}$	$\text {Im}\left( \theta \right)$	$(0.99\pm 1.98)10^{-2}$
$\text {Re}\left( \omega \right)$	$(1.09\pm 1.60)10^{-2}$	$\text {Im}\left( \omega \right)$	$\pm (6.40\pm 2.80)10^{-2}$
$S_{K_S}$	$-0.624\pm 0.030$	$R_{K_S}$	$\pm (0.781\pm 0.024)$
$C_{K_S}$	$\left( -1.44\pm 3.28\right) 10^{-2}$		
$S_{K_S}+S_{K_L}$	$(3.7\pm 4.9)10^{-2}$	$R_{K_S}+R_{K_L}$	$(-3.27\pm 4.3)10^{-2}$
$C_{K_S}-C_{K_L}$	$(-6.8\pm 6.3)10^{-2}$		
(II) Parameters – $\lambda _{K_S}+\lambda _{K_L}=0$ analysis			
$\text {Re}\left( \theta \right)$	$\pm (3.10\pm 1.51)10^{-2}$	$\text {Im}\left( \theta \right)$	$(0.14\pm 1.67)10^{-2}$
$\text {Re}\left( \omega \right)$	$(1.17\pm 1.59)10^{-2}$	$\text {Im}\left( \omega \right)$	$\pm (5.46\pm 2.70)10^{-2}$
$S_{K_S}$	$-0.640\pm 0.025$	$R_{K_S}$	$\pm (0.769\pm 0.022)$
$C_{K_S}$	$(1.61\pm 1.88) 10^{-2}$		

**Fig. 2:**
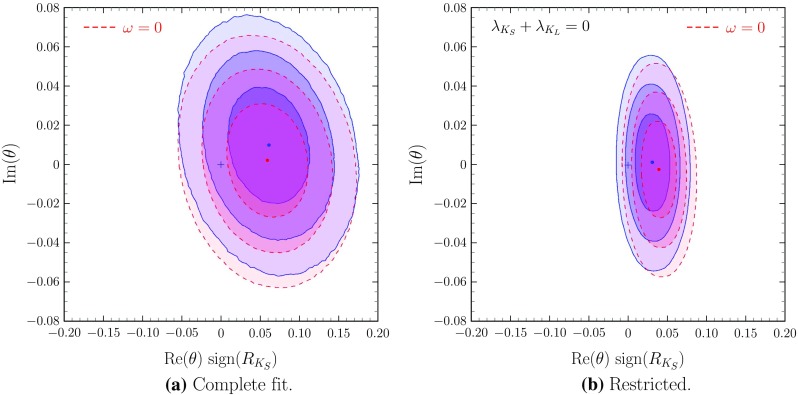
Comparison of the results with and without $\omega$; $\text {Im}\left( \theta \right)$ vs. $\text {Re}\left( \theta \right)$ regions are shown in different scenarios: blue regions with solid contours correspond to fits including the $\omega$-effect while red regions with dashed contours correspond fits without $\omega$, i.e. with $\omega =0$. Panel **a** shows the results for the general analyses while panel **b** shows the results for the analyses with $\lambda _{K_S}+\lambda _{K_L}=0$. **a** Complete fit. **b** Restricted

**Fig. 3:**
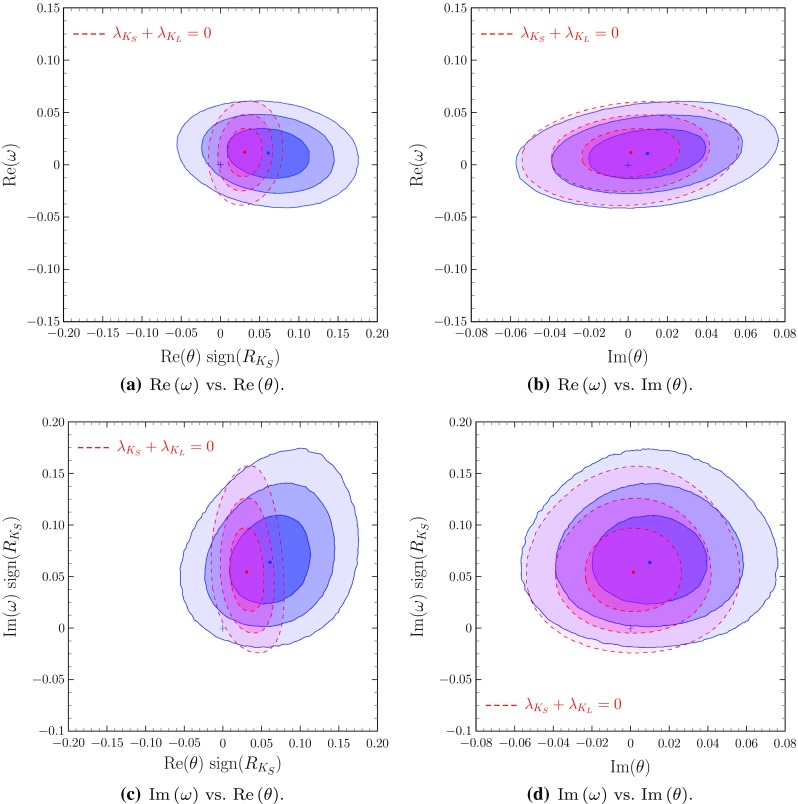
Correlations among $\omega$ and $\theta$. (Colour coding as indicated in Fig. 1.) **a** $\text {Re}\left( \omega \right)$ vs. $\text {Re}\left( \theta \right)$, **b** $\text {Re}\left( \omega \right)$ vs. $\text {Im}\left( \theta \right)$, **c** $\text {Im}\left( \omega \right)$ vs. $\text {Re}\left( \theta \right)$, **d** $\text {Im}\left( \omega \right)$ vs. $\text {Im}\left( \theta \right)$

In Table 1(I) we present the general result of the fit, whose most salient features are the following:

- Experimental data – more precisely the BaBar measurements in [2] – are sensitive for the first time to $\text {Im}\left( \omega \right)$, revealing a tantalizing $2.4\sigma$ deviation from $\text {Im}\left( \omega \right) =0$. These observables are also sensitive to $\text {Re}\left( \omega \right)$, but they do not show any significant deviation from $\text {Re}\left( \omega \right) =0$, and the previous determination $\text {Re}\left( \omega \right) =(0.8\pm 4.6)\times 10^{-3}$ [21] – using semileptonic channels – is still better than the present one.
- The results of the fit for the CPT violating parameter $\theta$ – in the evolution Hamiltonian – are compatible with the previous determination in [8] and the one performed by the BaBar collaboration in Ref. [29]. An exciting $2\sigma$ effect in $\text {Re}\left( \theta \right)$ is still present.
- The parameters that measure the presence of wrong flavour decays in $B_d\rightarrow J/\varPsi K$, i.e. $C_{K_S}-C_{K_L}$, $S_{K_S}+S_{K_L}$ and $R_{K_S}+R_{K_L}$, do not show any significant deviation from zero and the results are consistent with [8].
- In the case of $S_{K_S}$ and $R_{K_S}$ we observe that they differ by more than $1\sigma$ with respect to the determination in [8] without including the $\omega$ effect. Should this persist in the future, it could affect the precise determination of the unitarity triangle angle $\beta$.

In Table 1(II) we present the results of the same fit with the additional requirement of not having wrong flavour decays, $\lambda _{K_S}+\lambda _{K_L}=0$. No significant differences were noticed with respect to the conclusions discussed above for the general case. For completeness we show, when relevant, both analyses together in the same plots without further comments.

In Fig. 1 is shown the result for the new parameters not previously considered in the analyses where EPR entangled initial states where assumed. A deviation of the complex number $\omega$ from zero is found at 95% confidence level. This deviation comes essentially from $\text {Im}\left( \omega \right)$ and it represents a measurement of this parameter for the first time; the measurement of $\text {Re}\left( \omega \right)$ does not improve on the value obtained previously [21] from flavour specific decays.

The stability of the fitted value of the complex CPT violating parameter $\theta$ is shown in Fig. 2a, and b, where it is clear that the results for $\text {Re}\left( \theta \right)$ and $\text {Im}\left( \theta \right)$ do not change from the constrained case $\omega =0$ to the general case with arbitrary $\omega$. Cross correlations among the different components of $\theta$ and $\omega$ are shown in Fig. 3. For example, Fig. 3c shows the independence of $\text {Im}\left( \omega \right)$ and $\text {Re}\left( \theta \right)$: furthermore one can see in that figure that the point (0, 0) in this projection is at more than $2.5\sigma$ from the best fit values (or even at $3\sigma$ in the $\lambda _{K_S}+\lambda _{K_L}=0$ constrained analysis).

**Fig. 4:**
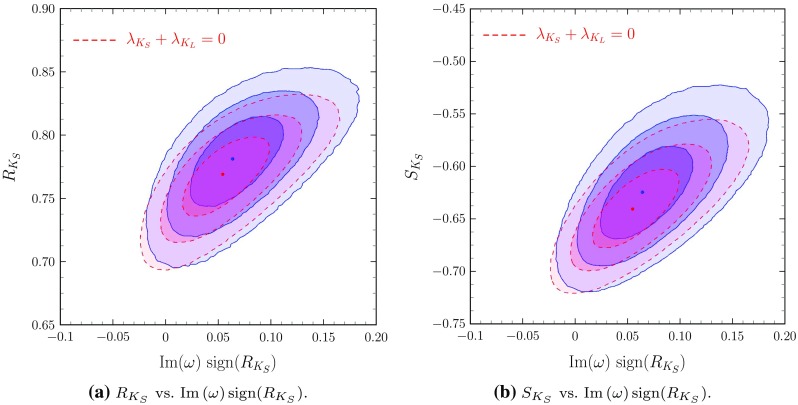
$R_{K_S}$ and $S_{K_S}$ vs. $\text {Im}\left( \omega \right)$; the presence of $\omega$ affects the extracted values of $R_{K_S}$ and $S_{K_S}$. (Colour coding as indicated in Fig. 1.) **a** $R_{K_S}$ vs. $\text {Im}\left( \omega \right) \text {sign}(R_{K_S})$, **b** $S_{K_S}$ vs. $\text {Im}\left( \omega \right) \text {sign}(R_{K_S})$

Finally, in Fig. 4, one can see the near linear correlation among $\text {Im}\left( \omega \right)$ and $R_{K_S}$. This explains why the presence of $\omega$ affects both $R_{K_S}$ and $S_{K_S}$.

**Fig. 5:**
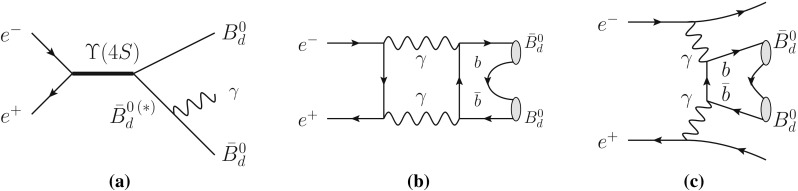
Potential background contributions to the $\omega$ effect

Before closing this section we would like to comment on an important issue that allows for the genuine CPTV $\omega$-effect to be disentangled from ‘fake’ effects that mimick $\omega$ and exist already within conventional Standard Model physics. The latter are background contributions associated with processes given in Fig. 5a–c, in which the $B^0_d\bar{B}^0_d$ pair is in a $C=+$ configuration. However, they have a $B^0_d\bar{B}^0_d$ invariant mass dependence that is distinct from that of the genuine $\omega$ effect, which involves an amplitude coherent with the $C=-$ state, and therefore corresponds to a resonant peak in the $B^0_d\bar{B}^0_d$ invariant mass distribution. A similar situation characterizes the entangled neutral kaon system [5].

In particular, the process of Fig. 5a associated with an intermediate $B^*$ state, has been discussed by Colangelo et al. [23], who performed a detailed calculation obtaining that the fake effect is of order $10^{-9}$, and concluded that a CPT test can be done at B factories [24].

The other two processes in Fig. 5b, c, are also expected to be very small; this can be seen, for example, for the absorptive part of Fig. 5b, by scaling the analog calculation in [25] to the $B^0_d$–$\bar{B}^0_d$ system. Similar processes have been analysed in $\phi$ factories for the $K^0$–$\bar{K}^0$ system [5, 26–28]. In general, these contributions are expected to be far below the present sensitivity.

## Conclusions

In the present article we have discussed the possibility of probing the entanglement-weakening CPT violating parameter $\omega$, that potentially signifies the breakdown of CPT operation as a result of quantum decoherence of matter in some models of quantum gravity, by means of identifying appropriate asymmetry parameters in the time evolution of intensities (14) between the two decays in a B factory, based on observables that have already been used in previous studies [8] probing independently T, CP and CPT symmetries in the absence of $\omega$. In the current analysis we have included, simultaneously with the $\omega$, also the conventional CPT parameter $\theta$, already considered in [8], which parameterizes CPT violation in the case of a well-defined CPT operator which, however, does not commute with the Hamiltonian of the system, indicating a violation of CPT parameterized within the framework of effective field theories (e.g. due to Lorentz symmetry violation by a space-time background), in contrast to the parameter $\omega$, which goes beyond that framework.

As we have demonstrated in the present article the set of observables of the B system (26), (27), (28) and (29) allow for a simultaneous determination (bounds) of the CPT violating parameters $\omega$ and $\theta$, which can thus be disentangled. The results obtained from the experimental data from the BaBar measurements [2] (see Table 1(I)) are sensitive for the first time to $\text {Im}\left( \omega \right)$, pointing towards a $2.4\sigma$ deviation from $\text {Im}\left( \omega \right) =0$, which we interpret as an upper bound. The observables (28), (29) are also sensitive to $\text {Re}\left( \omega \right)$, but they do not show any significant deviation from $\text {Re}\left( \omega \right) =0$, and in this sense they are inferior to the previous analyses [21] using equal sign semileptonic decay asymmetries of the B system, which yield $\text {Re}\left( \omega \right) =(0.8\pm 4.6)\times 10^{-3}$. The results (26) and (27) also allow a fit to the CPT violating parameter $\theta$, and they are compatible with the previous determination in [8] and the one performed by the BaBar collaboration in [29], pointing towards a $2\sigma$ effect in $\text {Re}\left( \theta \right)$, also interpreted as an upper bound for the corresponding parameter.

Moreover, the parameters that measure the presence of wrong flavour decays in $B_d\rightarrow J/\varPsi K$, i.e. $C_{K_S}-C_{K_L}$, $S_{K_S}+S_{K_L}$ and $R_{K_S}+R_{K_L}$, do not show any significant deviation from zero and the results are consistent with [8]. In the case of $S_{K_S}$ and $R_{K_S}$ we observe that they differ by more than $1\sigma$ with respect to the determination in [8] without including the $\omega$ effect. Should this persist in the future, it could affect the precise determination of the unitarity triangle angle $\beta$.

Before closing we stress once more that a quantum-gravity-decoherence-induced CPT violating and entanglement-weakening parameter $\omega$ may not only characterize the initial state of an entangled (neutral) meson system, but may also be generated as a result of a decoherening time evolution that goes beyond the local effective field theory framework [22]. A full analysis of that case will appear in a forthcoming publication.

## Footnotes

1 Another important aspect of the $\omega$ effect is its dynamical generation during a decoherence evolution, in which the particle interacts with its gravitational environment, for instance. As discussed in [22], a time-dependent contribution to the $\omega$ parameter may be generated in specific models of quantum decoherence, which could be present even if the initial state has an $\omega =0$. The relative magnitude of $\text {Re}\left( \omega (t)\right)$ and $\text {Im}\left( \omega (t)\right)$ in this case depends crucially on the decoherence space-time foam model used, but their generic form involves oscillatory dependences on time. Specifically, if one ignores conventional CPT violating effects, then the analysis of [22] has shown that the evolution of an entangled two-particle state contains in certain quantum space-time-foam models time-dependent $\omega (t)$ parts, which to leading order in appropriate small quantities assume the form

3 $$\begin{aligned}&|\psi \rangle \ni \mathrm{e}^{-i ( \lambda _0^{(1)} + \lambda _0^{(2)})t} \, \omega (t) (|k, \uparrow \rangle ^{(1)} \, |{-}k, \uparrow \rangle ^{(2)} {-} |k, \downarrow \rangle ^{(1)} \, |-k, \downarrow \rangle ^{(2)}), \quad \nonumber \\&\quad \omega (t) \sim \omega _0 \, \mathrm{sin}(2 |\varDelta E| \, t), \end{aligned}$$

which is purely generated by the evolution with no $$\omega $$ effect in the initial state $$t=0$$. In the above formula, the superscripts $$(i), i=1,2 $$ refer to individual particles, *k* is the momentum of the particle (assuming the decaying initial state to be at rest, for brevity), $$\varDelta E $$ is the energy difference between the appropriate single particle states, and the arrows denote the corresponding quantum numbers of a generic two state system, while $$\lambda _0$$ are the energy eigenvalues. The parameter $$\omega _0$$ in (3) is in general complex. In some concrete models of space-time quantum foam it could be purely imaginary [22]. In the present work we shall consider only constant $$\omega $$ in the initial state (2). We reserve details for the phenomenology of a time-dependent $$\omega $$-effect, generated during the evolution, for a future publication.

2 As is commonplace, subindices “H” and “L” correspond to the heavy and light $B_d$ states.

3 In the notation of Ref. [8], $\mathscr {C}_{h}[f,g]$, $\mathscr {C}_{c}[f,g]$ and $\mathscr {S}_{c}[f,g]$ are the $\omega \rightarrow 0$ limit of (respectively) $\mathscr {C}_{h}^\omega [f,g]$, $\mathscr {C}_{c}^\omega [f,g]$ and $\mathscr {S}_{c}^\omega [f,g]$ in Eqs. (15)–(17).

4 By construction $$C_{f}^2+S_{f}^2+R_{f}^2=1$$.

5 For small $\omega$ and $\theta$, these terms give the leading $\omega$ contributions: the $t_0$ dependence integrated over in Eq. (13) produces extra dilution factors $x/(1+(x/2)^2)^{-1}$ and $1/(1+(x/2)^2)^{-1}$ in Eqs. (15)–(17); fortunately, in the $B_d$ system, they do not thwart significantly the sensitivity to $\omega$.

6 Equation (14) gives the intensity *I*(*f*, *g*; *t*) expanded up to linear order in $\theta$ and $\omega$: the full result has indeed contributions that depend on $\text {Im}\left( \omega \right)$ and do not vanish when both *f* and *g* are flavour specific, but they have additional $\omega$ and/or $\theta$ suppressions which make them irrelevant. In any case, the actual fits in Sect. 3 are conducted using the full expressions.
